# Effect of Fibres on Physico-Mechanical Properties of Bulk-Fill Resin Composites

**DOI:** 10.3390/polym15163452

**Published:** 2023-08-18

**Authors:** Abdulrahman Alshabib, Nick Silikas, Hamad Algamaiah, Abdullah S. Alayad, Rahaf Alawaji, Shaikha Almogbel, Ahad Aldosari, Abdulaziz Alhotan

**Affiliations:** 1 Department of Restorative Dentistry, College of Dentistry, King Saud University, P.O. Box 60169, Riyadh 11545, Saudi Arabia; 2 Division of Dentistry, Faculty of Biology, Medicine and Health, The University of Manchester, Manchester M13 9PL, UK; 3 Dental Interns, College of Dentistry, King Saud University, P.O. Box 60169, Riyadh 11545, Saudi Arabiaahad.aldosari@gmail.com (A.A.); 4 Department of Dental Health, College of Applied Medical Sciences, King Saud University, P.O. Box 10219, Riyadh 12372, Saudi Arabia

**Keywords:** resin composites, fibre reinforcement, water storage, flexural strength, absorption, solubility

## Abstract

Objective: To measure the flexural strength (FS) of bulk-fill resin composites and assess their long-term water absorption and solubility properties with and without the inclusion of short glass fibres. Methods: One resin composite, everX Flow with fibres, and four commercially available bulk-fill composites without fibres, namely, PALFIQUE, Activa, SDR Plus, and Filtek Bulk Fill One, were tested. Six specimens (2 × 2 × 25 mm) were fabricated for each material and stored in water for 1 day and 30 days to measure the flexural strength using a three-point bending test. To evaluate water absorption and solubility, circular disks measuring 15 × 2 mm (n = 5) were immersed in water for 60 days, and their weights were recorded periodically. After 60 days, the specimens were dried for an additional 21 days to determine solubility. Results: Flexural strength values ranged from 101.7 to 149.1 MPa. Significant distinctions were observed among the resin composites at the onset of the study (*p* < 0.05). The highest FS value was identified in everX Flow, while ACT exhibited the lowest (*p* < 0.05). However, the flexural strength values exhibited a significant decrease with increased storage time (*p* < 0.05), except for ACT, which demonstrated a noteworthy increase. Concerning water absorption and solubility, ACT displayed the highest absorption, while the range of solubility varied from −0.88 to 5.8 μg/mm^3^. ACT also had the highest solubility, whereas everX Flow exhibited negative solubility. Significance: The addition of short fibres, along with potential differences in matrix composition, enhanced the flexural strength of everX Flow. However, the substantial reduction in flexural strength observed in everX Flow and SDR following exposure to water corroborates the manufacturers’ recommendation to apply a conventional resin composite cap on these materials.

## 1. Introduction

Resin composites have been utilized in clinical practice for nearly five decades, and during this time, specific “developmental cycles” have been introduced to address any shortcomings identified in clinical settings. During the early stages of resin composite use, in the 1980s and 1990s, there was a significant emphasis on enhancing particulate filler systems, which resulted in the development of materials with enhanced wear resistance and superior mechanical properties [1]. In the subsequent decade, efforts were invested in reducing polymerisation shrinkage to overcome concerns such as the formation of interfacial gaps, postoperative sensitivity, and cuspal deflection [2].

More recently, the convenience offered by bulk-fill composites has led to their increased popularity [3]. This approach allows dental practitioners to restore larger cavities in a single increment, saving valuable time during treatment process. These materials have been investigated extensively in vitro, and several clinical studies have been performed, usually with a follow-up of one or two years [4,5]

The durability and performance of resin composite restorations are heavily influenced by their ability to stand up to the conditions associated with diverse and harsh intraoral environments, which vary among patients in terms of masticatory forces, occlusal habits, temperature fluctuations, bacteria, abrasive foods and salivary enzymes. These factors heavily influence the longevity and performance of resin composite restorations [6,7,8,9]. Additionally, the performance of resin composites is significantly influenced by a variety of factors, including the crosslink density, porosity, hydrophilicity, quality, nature of the filler system, and other characteristics of the resin composite network [9,10,11].

Intraoral deterioration is one of the primary reasons for the failure of direct restorations, which can adversely impact the wear resistance, microhardness, dimensional stability, colour stability, and fracture resistance of resin composites [9,12]. Moreover, the characteristics of resin composites are greatly affected by factors like water absorption, temperature fluctuations, and the duration of exposure to aquatic media [13]. Other chemicals present in the oral environment, such as acidic substances found in certain foods and beverages, can also interact with the resin matrix and potentially lead to degradation. Additionally, the by-products of acidogenic bacteria under restoration margins can further hinder the lifespan and effectiveness of resin composites [14].

Water, known for its polar nature and relatively small molecule size compared to polymer chains, exhibits effective solvation properties. When a polymer matrix comes into prolonged contact with water, water molecules diffuse into the matrix, aided by their polarity and size [15]. In the case of dimethacrylates, water penetrates the polar polymer matrix, the intermolecular interactions between the polymer chains can weaken, resulting in improved matrix flexibility [15]. This water-induced plasticisation can cause dimensional changes, such as straightening, within the polymer chain, leading to material swelling. The extent of these effects within the crosslinked network depends on the utilized monomers’ hydrophilic properties and the substance’s inter-polymer pressures [16].

The impact of solvents on dental composites can vary, as conflicting findings have been reported in the literature. For example, some studies have reported significantly reduced flexural strength when dental composites are immersed in water for 1 or 2 months [17]. Similarly, water aging has been associated with a decrease in fracture toughness of 10–35% [7,18]. However, different studies have yielded contradictory results regarding the effect of water on the flexural strength or fracture toughness of composites [19,20].

These discrepancies can be attributed to various factors, including changes in the resin composites’ composition and variations in testing methodologies. For example, Tanaka et al. observed a significant reduction of 30% in compressive strength, diametral tensile strength, flexural strength, and elastic modulus of traditional composites after water storage. However, a similar investigation on an experimental composite containing a fluorinated polymer showed a decrease only in flexural strength. This highlights the significant role resin components can play in determining the solvent resistance of dental composites [21].

In this study, the flexural strength of four commercially available bulk-fill resin composites was compared to that of one fibre-reinforced resin composite to evaluate the impact of water and analyse the time-dependent water sorption and associated features of fibre-reinforced resin composites. The null hypotheses are:

- There will be no difference in flexural strength among the materials.
- There will be no difference in flexural strength among the materials after 1 and 30 days of water storage at 37 °C.
- There will be no difference in water sorption or solubility after 140 days of water exposure.

## 2. Materials and Methods

Table 1 displays the compositions, shades, and lot numbers of the bulk-fill materials used in the study, which include one short fibre-reinforced composite, everX Flow (GC), and four commercially available bulk-fill composites without fibres, namely PALFIQUE (Tokuyama), Activa (Pulpdent), SDR Plus (Dentsply Sirona) and Filtek Bulk Fill One (3M).

### 2.1. Fibre Length Measurement

The following methodology was employed to extract and characterise the inorganic components from everX Flow. The procedure for preparing the specimen and determining the lengths of fibres was as follows. The composite paste, weighing 0.5 g, was placed in a glass jar as the initial step. To facilitate mixing, 20 mL of 99.9% pure tetrahydrofuran (THF) was added to the composite paste. The mixture was thoroughly swirled for two minutes using a spatula. Subsequently, the mixture was divided into two equal portions and transferred to separate tubes. To ensure complete evaporation of the THF solvent, the inorganic component was transferred to plastic tubes and subjected to drying in an EZ-2 Elite solvent evaporator at 60 °C for three hours.

For the characterisation of the inorganic components, a specimen was prepared through a vacuum sputter-coating process. The specimen was coated with a 60/40 Au/Pd alloy with a layer thickness of 10 nm. This coating was achieved by applying vacuum pressure for two minutes. The coated specimen was then subjected to scanning electron microscopy (SEM) analysis using a Quanta 650 FEG SEM FEI Company, Hillsboro, OR, USA) to obtain high-resolution images of the glass fibres. The acquired images of the specimen were then processed using ImageJ software (version 1.8.0). Specifically, the software was utilised to accurately measure the lengths of 50 randomly selected fibres from the obtained images. This ensured precise and reliable measurements for the fibre lengths.

### 2.2. Flexural Strength and Modulus Measurement

For the three-point bending test, the following steps were performed. Eight bar-shaped specimens were fabricated from each resin composite, with dimensions of 25 × 2 × 2 mm^3^ (Table 1), following the respective manufacturers’ instructions and ISO 4049 specifications [22]. As mentioned above, the resin composite samples were packed inside a Teflon mould, which was squeezed between two clear Mylar strips and glass slides (1 mm thick). Three overlapping areas of irradiation were applied along the length of the specimens from the top using an LED curing unit. The measured average tip intensity of the unit was 1200 mW/cm^2^ (Elipar S10, 3M Espe, Seefeld, Germany) and the irradiation time was 20 s. After removing the specimens from the mould, both sides were gently finished using sandpaper. The finished specimens were stored in distilled water at 37 ± 1 °C for 24 h to ensure water immersion. Each specimen was placed under the pressure of a central load in a three-point bending test using a universal testing machine (Instron, Universal testing machine, Instron Corp, Norwood, MA, USA). The crosshead speed was set at 0.5 mm/min, and a load cell with a capacity of 5 kN was utilized. The span length between the supports was 20 mm, and an indenter with a diameter of 2 mm was used. The test continued until the fracture of the specimen point was reached.

The flexural strength ($O_{f}$) was calculated using the following formula [23]:

(1) $$O_{f}=\frac{3\;FmI}{2\;{bh}^{2}}$$

where *Fm*: the applied load (N) at the highest point of the load–deflection curve; *I*: the span length (20 mm); *b*: specimen width; *h*: specimen thickness.

The elastic modulus was determined by calculating the slope of the linear region of the load–deflection curve using the following equation [24,25]:

(2) $$E_{f}=\frac{L^{3}F}{4wh^{3}d}$$

where *w* is the width (mm) and *h* is the height (mm) of the specimen, *L* (mm) is the distance between the supports, and *d* (mm) is the deflection due to load *F* (N) applied at the middle of the specimen.

### 2.3. Water Sorption and Solubility

#### 2.3.1. Specimen Preparation

Teflon moulds were used to fabricate six disc-shaped specimens for each material. The dimensions of the moulds were 15 × 2 mm. The moulds were placed between two sections of clear Mylar strip, with glass slides with a thickness of 1 mm placed on each side. The resin was cured using an LED curing unit. The average tip irradiance was 1.2 W/cm^2^ (as previously described). Each specimen was irradiated for 20 s on five sections of the top surface using the light cure unit. After the curing process, the specimens were carefully removed from the moulds, and 1000-grit silicon carbide paper was used to smooth any rough edges. Following 24 h of storage, a precision-calibrated balance was used to weigh the specimens with an accuracy of ±0.01 mg (BM-252, A&D Co., Tokyo, Japan). The full weighing process was repeated until a constant mass (m_1_) was achieved.

Using a digital calliper, the mean diameters of the specimens were measured at three different locations, and their thicknesses were measured at five different locations: the centre and four evenly spaced points around the circumference. These measurements were used to calculate each specimen’s volume (V) in cubic millimetres using Equation (3), where r represents the circumference of the circular disc and h represents the disc’s height (mm).

(3) $$V=\pi r^{2}h$$

where r is the circular disc’s radius in millimetres and h is the disc’s height in millimetres.

#### 2.3.2. Sorption and Solubility Measurement

For water sorption and solubility testing, six specimens from each group were placed in separate glass containers, each containing 15 mL of distilled water, and maintained at 37 ± 1 °C for 60 days. Daily weighing was conducted during the first week, followed by measurements at 14, 21, 28, and 60 days, which marked the attainment of water sorption equilibrium. At each time point, the specimens were carefully removed from the water using tweezers, dried with hand towels until no moisture was present, air dried for 15 s, and then weighed one minute later. After each measurement, fresh distilled water was added to the containers to maintain a constant water pH. The mass measurements at different time points were denoted as m_2_(t), where t indicates the specific time.

After completing the 60-day water sorption weighing process, the specimens underwent reconditioning in a desiccator following the previously described procedure. At intervals of Day 1, 7, 14, and 21, the mass of the specimens at these testing sites was recorded as m_3_(t). By Day 21, a consistent mass was achieved.

To determine water sorption (W_sp_) and solubility (W_sl_) for each specimen in each group, Equations (4) and (5), respectively, were used. In these equations, m_1_ represents the conditioned mass of the specimen in micrograms (μg), m_2_ represents the mass of the specimen after water immersion (μg), m_3_ represents the reconditioned mass of the specimen (μg), and v represents the volume of the specimen in cubic millimetres.

(4) $$w_{sp}=\frac{m_{2(t)}-m_{3}}{v}$$

(5) $$w_{sl}=\frac{m_{1}-m_{3(t)}}{v}$$

where m_1_ is the conditioned mass (μg) of the specimen, m_2_ is the mass (μg) of the specimen after immersion in water, m_3_ is the mass (μg) of the reconditioned specimen and v is the volume (cubic millimetres) of the specimen.

Finally, the percentage solubility (Md%) and the percentage mass change (Ms%) were calculated using Equations (6) and (7), respectively. These values indicate the overall mass loss of the components.

(6) $$Sorption\;mass\;change\left(M_{s}\%\right)=\frac{m_{2\left(t\right)}-m_{1}}{m_{1}}\times 100$$

(7) $$Desorption\;mass\;change\left(M_{d}\%\right)=\frac{m_{3\left(t\right)}-m_{1}}{m_{1}}\times 100$$

### 2.4. Statistical Analyses

Statistical software (SPSS Inc version 27, Chicago, IL, USA) was used to conduct the statistical analyses for this study. The normality of each variable was assessed using the Shapiro–Wilk test. The mean and standard deviation were calculated for the variables. Parametric analysis was conducted as the variables exhibited a normal distribution. To find any variations in flexural strength and flexural modulus (independent variables) between the materials in terms of storage time (*p* ≤ 0.05), two-way ANOVA was used, followed by Tukey post hoc testing (*p* ≤ 0.05). An independent *t*-test was also utilized to determine the effect of the differences in each storage time (1 d and 30 d comparison) for each material (*p* ≤ 0.05).

One-way ANOVA was also performed at a significance level of *p* ≤ 0.05 to ascertain the significance of any variations between the materials in terms of water sorption after 60 days, and mass change. A Tukey post hoc test was then conducted to identify any significant differences that could be observed within the materials. The same statistical analysis was performed to assess the solubility and mass loss differences between the samples by evaluating weight changes after the 21-day desorption cycle.

## 3. Results

### 3.1. Fibre Length Measurement

The measured length of the glass fibres in the everX Flow composite ranged from 218 µm to 811 µm, with an average length of 418 µm. Among the fibres, 68% fell within the length range of 200–400 µm, with an average length of 340 µm. The remaining 32% of the fibres ranged between 500–811 µm, with an average length of 619 µm. The results of the length distribution of glass fibres in the everX Flow composite are summarized in Table 2.

### 3.2. Flexural Strength

Table 3 displays the means of flexural strength for each resin composite after Day 1 and Day 30 of storage in distilled water at 37 °C. The statistical analysis revealed a statistically significant difference in the flexural strength of the materials subjected to the flexural strength tests (*p* < 0.05). In addition, the independent *t*-test indicated that there was a statistically significant difference in the flexural strength of the tested material between Day 1 and Day 30 of storage in distilled water at 37 °C. However, the information does not provide specific values for the means and standard deviations.

### 3.3. Flexural Modulus

Table 4 presents the means of flexural modulus for each resin composite after Day 1 and Day 30 of storage in distilled water at 37 °C. The statistical analysis revealed a statistically significant difference in the flexural modulus of the tested materials (*p* < 0.05). However, the outcomes of the independent *t*-test indicated no statistically significant variations in the flexural modulus of the tested materials between Day 1 and Day 30 of storage in distilled water at 37 °C. It is important to note that the data do not include specific values for the means and standard deviations.

### 3.4. Water Sorption and Solubility

Table 5 presents the means and standard deviations of sorption and solubility values for the resin composites. At Day 60, the water sorption of the materials ranged from 18.4 to 29.2 µg/mm^3^. The largest mass of water uptake, with a value of 1.60%, was observed in the EXF specimens. Throughout the immersion period, a gradual increase in the mass of water uptake in all tested materials was observed, until it reached equilibrium on Day 60. During the desorption cycle, the mass of the materials rapidly fell and eventually achieved a constant mass by Day 21. On Day 60, no significant variations in water sorption were observed between the different materials. However, significant variations in water sorption were recorded between the specimens in the PAL and FBF materials and all the tested materials (*p* ≤ 0.05).

**Figure 1:**
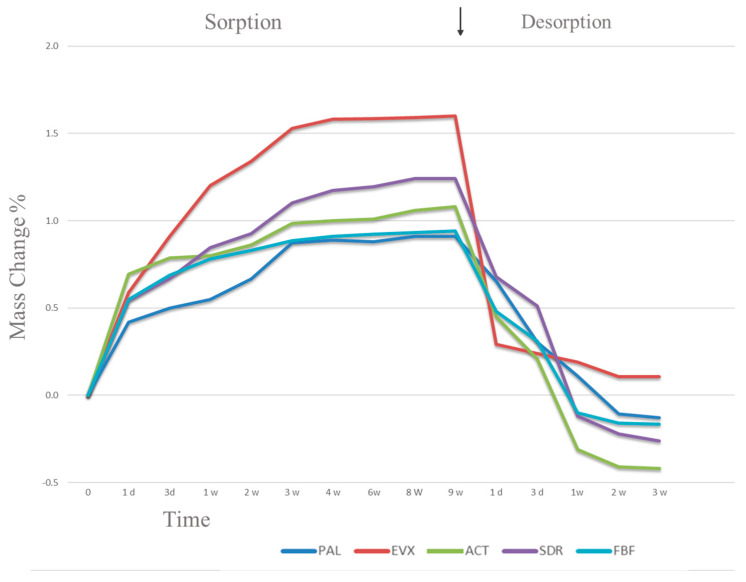
Mass change percentage.

Figure 1 displays the percentage mass change in the tested resin composite during the water sorption/desorption cycle. It is evident that all the composites showed an increase in mass due to water uptake before reaching an equilibrium state 60 days later. It is worth noting that the initial mass (m_1_) of each composite was generally higher than its reconditioned mass (m_3_), except for EVF, which exhibited a lower initial mass compared to its reconditioned state.

After a duration of 60 days, the water sorption levels ranged between 18.4 and 29.2 μg/mm^3^, as presented in Table 4. Among the composites, ACT displayed the highest sorption, followed by SDR and EVF, which exhibited similar levels. Conversely, both FBF and PAL materials exhibited lower water sorption levels, with no statistically significant differences being observed between them (*p* ≥ 0.05).

Regarding solubility, the resin composites exhibited ranging from −0.88 to 5.8 μg/mm^3^, as shown in Table 5. ACT displayed the highest solubility levels compared to the other tested materials. Notably, EVF demonstrated a negative solubility value (−0.88 μg/mm^3^).

### 3.5. SEM Examination

**Figure 2:**
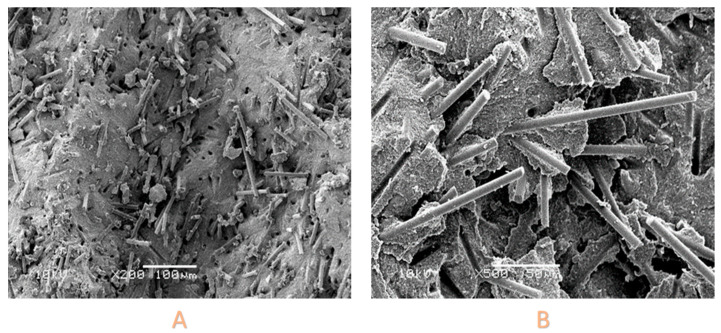
(**A**) Flexural strength specimen (×200 magnification) showing random orientation of fibres. (**B**) flexural strength specimen (×500 magnification); showing pull-out of the fibres. Resin clusters are observed in both images.

**Figure 3:**
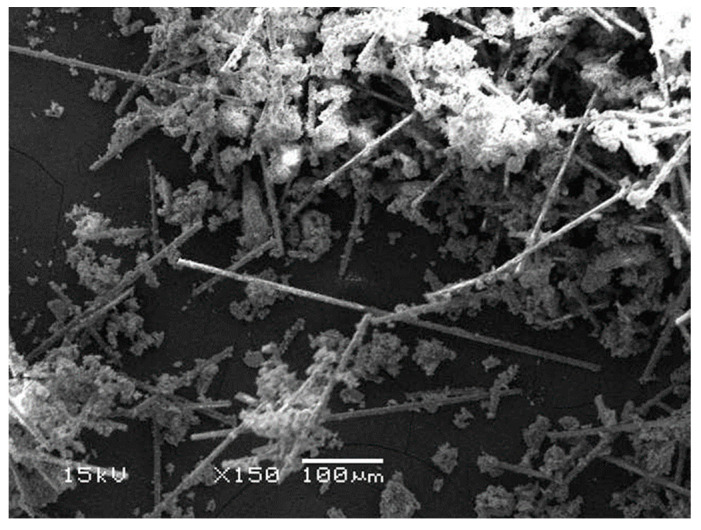
SEM images (×150) of extracted glass fibres from fibre-reinforced resin composite.

A representative scanning electron microscopy (SEM) image of EVF was examined, and the findings are presented in Figure 2A,B. These figures reveal the occurrence of fibre pull-out and fibre breakage at the fracture point. Furthermore, in Figure 3, which was captured at a magnification of ×150, the presence of an extracted glass fibre can be observed. Notably, the fibres exhibited an average length of 418 μm. The SEM micrographs clearly demonstrate the combination of particulate fillers and fibres within the EVF material.

## 4. Discussion

### 4.1. Flexural Strength

For many years, secondary caries have been widely documented as the primary factor contributing to the failure of posterior resin-composite restorations [6]. However, emerging research has revealed that the bulk fracture of the composite filling is, in fact, the most common cause of replacement for posterior restorations within the first five years of use [26]. A comprehensive meta-analysis on posterior resin-composite restorations revealed that at least 5% of restorations experience material fracture within a ten-year period [27].

Flexural strength (FS) is a valuable and frequently employed measurement that provides meaningful insights into a material’s resistance to fracture [28]. The first part of the current study aimed to investigate the influence of different resin composite materials and water storage conditions on the flexural strength (FS) of various restorations. The flexural strength (FS) of the tested materials was initially assessed at baseline (24 h) and ranked in descending order as follows: EVF, FBF, PAL, SDR, and ACT. Subsequently, after 30 days of water storage, variations in material stability behaviour were observed compared to the baseline values. In particularly, EVF demonstrated a significant reduction in flexural strength. The findings demonstrated that both the material type and water storage have significant effects on FS (*p* < 0.05). Consequently, the first and second null hypotheses were rejected, confirming the notable impact of material composition and environmental conditions on the flexural strength of resin-composite restorations. According to the method of application of the resin composite evaluated in the current study, the composites can be categorized into one of two groups: (1) bulk-fill composites that should be used clinically with a capping layer (EVF, SDR), with EVF having the highest values of 149.1 MPa compared to 110 MPa for SDR; and (2) composites that can be used clinically without a capping layer (FBF, PAL, and ACT), with values of 139.8, 137.7 and 108.6 MPa, respectively.

Enhanced resistance to crack propagation was observed in EVF. This can be attributed to the favourable properties of its fibre and matrix components. Notably, EVF incorporates longer fibres that surpass the critical fibre length [29,30], facilitating more efficient stress transfer from the matrix. These findings align with existing literature in the field [7,31,32].

However, it is important to highlight that a significant decrease in flexural strength (FS) of 24% was observed following 30-day immersion in water. This decline in FS can primarily be attributed to hydrolytic degradation occurring between the matrix and the glass fibres. It should be noted that glass-fibre-reinforced composites generally exhibit higher water absorption than compared to composites reinforced with particulate fillers [10].

A toughening effect may occur due to the aqueous plasticisation of the resin matrix [17], which could explain the increased strength observed in ACT after 30 days of storage, as evidenced by the reduction in elasticity of 32%. During testing, an interesting observation was made with Activa; specifically, the specimen exhibited a noticeable bend when subjected to load before fracture. This characteristic is a result of the material’s elastic modulus. It is worth noting that the elastic modulus plays a significant role in determining flexural strength [28].

In the context of dental composites, it is important to avoid low modulus of elasticity values, as they result in high levels of distortion. Similarly, high flexural strength should also be approached with caution in materials that exhibit excessive flexibility (low modulus of elasticity), because this compromises their overall strength. In such cases, load distribution becomes uneven, leading to a lack of horizontal distribution of chewing forces across the periodontium. Consequently, the surface of the restoration is subjected to high tensile loads due to occlusal pressure, potentially impacting the adhesion to the tooth structure. Additionally, occlusal loads acting on flexible filling materials within the cavity can induce lateral expansion, which may ultimately cause fractures in the tooth surface. It is crucial to consider these factors to ensure the optimal performance of dental composites.

There is a direct correlation between the flexural strength of resin-based composite (RBC) materials and the filler content [33]. This observation elucidates the significant disparity in mean flexural strength between FBF, which has a filler content of 76.5 wt.%, and SDR, which has a filler content of 68 wt.%. However, it should be noted that PAL, with a filler content of 70 wt.%, exhibited lower flexural strength than FBF but higher than SDR. Additional factors, such as the composition of the resin matrix, might also contribute to the mechanical properties, including flexural strength, of RBC materials [34,35].

The resin matrix of FBF comprises aromatic urethane dimethacrylate (AUDMA), which is a high-molecular-weight monomer that minimizes the presence of reactive groups in the resin chain. This characteristic enhances the rigidity of the final polymeric matrix during the polymerisation process [36]. Furthermore, the incorporation of addition–fragmentation chain transfer monomers (AFM) in FBF can improve the homogeneity of the polymer network, potentially leading to enhanced mechanical properties [37]. Recent studies have found that FBF offers superior flexural strength [38,39]. This can be attributed to both its filler content and the composition of the resin matrix. These findings underscore the combined influence of filler content and resin matrix composition on the flexural strength and mechanical properties of RBC materials.

### 4.2. Sorption and Solubility

The subsequent phase of this study aimed to evaluate the water sorption and solubility properties of several resin composites during nine weeks of immersion in water. The results revealed substantial variations among the tested materials, leading to the rejection of the third and fourth null hypotheses. According to ISO Standard 4049 [22], which sets a maximum sorption limit of 40 μg/mm^3^ and a solubility threshold of less than 7 μg/mm^3^ after 7 days of storage, all composite materials met the specified criteria, even after an extended period of water sorption. This indicates that the aqueous challenge employed in this study was more rigorous than the standard seven-day challenge described in the ISO protocol.

The water sorption and solubility behaviours of resin composites are primarily influenced by their hydrophilic properties and the extent of crosslinking in the network structure. Additionally, the degree of porosity in the material and the nature of the filler matrix significantly impact the amount of solvent absorbed during the exposure period [40,41].

The findings of the current study revealed a negative correlation between the sorption values and the filler loading, with the exception of EVF. This observation aligns with previous studies [10,42] that have reported similar results. As the weight percentage of filler increases, the wt.% of the polymeric matrix decreases, leading to a decrease in water sorption. Although glass fillers, whether in particulate or fibre form, are generally considered to have minimal impact on the sorption process, it is still plausible that water molecules can adsorb onto their surface. The occurrence of this adsorption depends on the integrity of the interface between the resin matrix and the glass fillers [43].

Fibre-reinforced composites, similar to particulate-filled composites, exhibit a degradation in properties when exposed to water. Water diffusion occurs primarily through the resin matrix. Areas with inadequate impregnation of fibres are more susceptible to water uptake [43]. Water sorption is influenced by multiple factors, including the hydrophilicity of the resin matrix, the amount of the inorganic phase (fibres and particulates), and the quality of silanisation. Furthermore, the capillary action of fibres can contribute to increased water absorption, resulting in mass gains [44]. In cases where glass fibres are exposed during finishing and polishing procedures, water sorption along the fibre–resin interface surpasses diffusion through the polymer matrix. This capillary effect is particularly evident in e-glass fibre-reinforced composites. Additionally, polymerisation shrinkage can lead to the formation of capillaries between the resin matrix and the glass fibres, especially in resin systems with high polymerisation shrinkage, such as TEGDMA and Bis-EMA monomers. It is worth noting that the negative solubility observed in the EVF material might be attributed to incomplete dehydration rather than indicating insolubility. This suggests that the solubility of EVF is relatively low. Another possible explanation is that hydrolytic chemical reactions occur between metal oxides, glass fillers, and water, formatting the metal hydroxides within the composite material [45].

The current study’s findings indicate that EVF, SDR, and ACT resin composites exhibited different behaviour from that of the other materials, and demonstrated the highest sorption values. Per the Instructions for Use, these materials (excluding ACT) are intended for dentin replacement and should be covered with a conventional particulate-filled composite. However, there may be certain clinical situations where this procedure is not feasible [46]. It is important to note that ACT is a dual-cured material that can be used without a capping layer. However, the current study revealed that ACT exhibited the highest sorption and solubility values. This observation can be attributed to its low filler content and the use of a modified polyacrylic acid that may not form a stable acid in the presence of water. Based on these findings, it is advisable to either cap this material or avoid placing it in areas subject to high occlusal loading.

This study evaluated the effect of incorporating short glass fibres in bulk-fill resin composites, which lead to notable enhancements in flexural strength, while also highlighting challenges related to reduced durability under prolonged water exposure compared to the non-fibre-reinforced materials. Addressing the mechanisms underpinning fibre–matrix interactions, optimising composite formulations, and conducting rigorous clinical trials will be imperative to advance the practical implementation of these novel materials in dental applications.

The results presented in this study were obtained under controlled laboratory conditions. However, it is important to acknowledge that clinical conditions are different, and various factors, such as insertion and handling, can affect the physical and mechanical properties and in vivo performance of the materials.

## 5. Conclusions

The conclusions drawn from the results of the current study are as follows:

- The glass-fibre-reinforced resin composite (EVF) demonstrated the highest flexural strength (FS) among the tested materials, while SDR and ACT exhibited the lowest FS.
- The manufacturers recommend applying a conventional resin composite cap on EVF and SDR. The considerable decrease in FS observed in EVF and SDR following exposure to water supports this recommendation.
- While variations were apparent in the water sorption and desorption cycles of the resin-matrix composites investigated in the current study, all the tested materials complied with the ISO 4049 requirements for water solubility and sorption, irrespective of the length of the sorption period.

## 6. Clinical and Practical Significance

- The enhanced mechanical properties of EVF introduce possibilities for innovative dental restorations in situations demanding superior strength and durability.
- Insights into water exposure effects enrich the understanding of material behaviour in realistic oral conditions, guiding informed material selection and clinical practice.

## Figures and Tables

**Table 1:** Composition of materials according to the manufacturer’s published data.

Material		Manufacturer	Type	Filler Load wt%	Filler Type	Resin Matrix
Code	Name					
EVF	ever X Flow	GC Corporation, Tokyo, Japan	Flowable Fibre reinforced BulkFill	70%	E-Glass short fibres, Barium Borosilicate Glass particulate	Bis-GMA, TEGDMA, Bis-EMA
ACT	Actvia	Pulpdent	Dual cure bulk fill	55.4%	Reactive ionomer glass fillers and sodium fluoride	Patented ionic resin matrix, shock-absorbing rubberised resin (diurethane and other methacrylates with modified polyacrylic acid)
PAL	PALFIQUE Bulk Flow	Tokuyama Dental, Tokyo, Japan	Flowable bulk fill	70%	Zirconia filler Silica fillers/	Bis-GMA, TEGDMA, Bis-MPEPP, Mequinol, Dibutyl hydroxyl toluene
FBF	Filtek Bulk fill	3M ESPE, St. Paul, MN, USA	Nano-hybrid Bulk fill A2 shade	76.5%	ytterbium tytterbium trioride and zircon silica	DDDMA, UDMA, AUDMA
SDR	SDR Flow composite	Dentsply Sirona Charlotte, NC, USA	Bulk fill	68%	Bari-um-alumino-fluoro-borosilicate glass and strontium alumino-fluoro-silicate glas	Modified UDMA, ethoxylated ethoxylated Bisphenol A dimethacrylate, and TEGDMA

Bis-GMA: Bisphenol-A glycidyl dimethacrylate; TEGDMA: Triethylene glycol dimethacrylate; Bis-MPEPP: Bisphenol A polyethoxy methacrylate; UDMA: Urethane dimethacrylate; AUDMA: aromatic urethane dimethacrylate; DDDMAA: 1, 12-Dodecanediol dimethacrylate; AFM: addition fragmentation monomer; Bis-EMA: Bisphenol-A ethoxylated dimethacrylate.

**Table 2:** Glass fibre lengths.

	Fibre Length	
	200–400 µm	400–811 µm
Fibre lengths grouped by average length (mm)	340	619
Fibre lengths grouped by percentage values (%)	68%	32%

**Table 3:** Flexural strength of resin composites.

Material	Flexural Strength (MPa)		
	1 Day	30 Days	Change %
EXF	149.1 (8.1) ^Aa^	111.6 (5.4) ^Ab^	−24
PAL	137.7 (12.0) ^A^	126.5 (9.3) ^B^	−8
FBF	139.8 (9.3) ^A^	119.6 (10.1) ^B^	−13
SDR	110.0 (7.1) ^Bc^	88.3 (7.2) ^Cd^	−20
ACT	108.6 (10.4) ^B^	129.5 (9.6) ^B^	+11

In each column, different superscript uppercase letters indicate significant differences between materials (*p* ≤ 0.05). For each row, different superscript lowercase letters indicate significant differences between exposure times (1 day and 30 days) within a material (*p* ≤ 0.05).

**Table 4:** Flexural modulus of resin composites.

Material	Flexural Modulus (GPa)		
	1 Day	30 Days	Change %
EXF	11.9 (1.4) ^Aa^	10.8 (1.1) ^Aa^	−9
PAL	10.7 (0.9) ^Aa^	9.9 (1.0) ^Aa^	−8
FBF	9.8 (0.8) ^Aa^	8.1 (0.3) ^Aa^	−17
SDR	7.1 (0.2) ^Bb^	5.9 (0.5) ^Bc^	−17
ACT	6.2 (0.3) ^Cd^	4.2 (0.8) ^Ce^	−32

In each column, different superscript uppercase letters indicate significant differences between materials (*p* ≤ 0.05). For each row, different superscript lowercase letters indicate significant differences between exposure times (1 d and 30 d) within a material (*p* ≤ 0.05).

**Table 5:** Water sorption (μg/mm^3^) and mass change % of resin composites.

Material	Sorption after 60 Days and Solubility after 21 Days		
	% Mass Change	Sorption	Solubility
EXF	1.60 (0.19) ^A^	26.9 (1.8) ^A^	−0.88 (0.10) ^A^
PAL	0.91 (0.11) ^B^	18.4 (2.0) ^B^	2.8 (0.19) ^B^
FBF	1.12 (0.10) ^C^	21.89 (2.1) ^B^	3.1 (0.59) ^B^
SDR	1.24 (0.21) ^D^	28.11 (2.5) ^A^	3.2 (0.37) ^B^
ACT	1.08 (0.18) ^C^	29.2 (3.8) ^A^	5.8 (0.31) ^C^

In each column, different superscript uppercase letters indicate significant differences between materials (*p* ≤ 0.05).

## Data Availability

The data presented in this study are available on request from the corresponding author.
